# Antibodies against Angiotensin II Type 1 and Endothelin 1 Type A Receptors in Cardiovascular Pathologies

**DOI:** 10.3390/ijms23020927

**Published:** 2022-01-15

**Authors:** Giovanni Civieri, Laura Iop, Francesco Tona

**Affiliations:** Department of Cardiac, Thoracic, Vascular Sciences and Public Health, University of Padova, 35128 Padova, Italy; giovanni.civieri@yahoo.it (G.C.); laura.iop@unipd.it (L.I.)

**Keywords:** autoantibodies, angiotensin, endothelin, receptors, ETAR, AT1R, cardiovascular, preeclampsia, transplantation, coronary

## Abstract

Angiotensin II receptor type 1 (AT1R) and endothelin-1 receptor type A (ETAR) are G-protein-coupled receptors (GPCRs) expressed on the surface of a great variety of cells: immune cells, vascular smooth cells, endothelial cells, and fibroblasts express ETAR and AT1R, which are activated by endothelin 1 (ET1) and angiotensin II (AngII), respectively. Certain autoantibodies are specific for these receptors and can regulate their function, thus being known as functional autoantibodies. The function of these antibodies is similar to that of natural ligands, and it involves not only vasoconstriction, but also the secretion of proinflammatory cytokines (such as interleukin-6 (IL6), IL8 and TNF-α), collagen production by fibroblasts, and reactive oxygen species (ROS) release by fibroblasts and neutrophils. The role of autoantibodies against AT1R and ETAR (AT1R-AAs and ETAR-AAs, respectively) is well described in the pathogenesis of many medical conditions (e.g., systemic sclerosis (SSc) and SSc-associated pulmonary hypertension, cystic fibrosis, and allograft dysfunction), but their implications in cardiovascular diseases are still unclear. This review summarizes the current evidence regarding the effects of AT1R-AAs and ETAR-AAs in cardiovascular pathologies, highlighting their roles in heart transplantation and mechanical circulatory support, preeclampsia, and acute coronary syndromes.

## 1. Introduction

The role of autoantibodies against angiotensin-II-type-I and endothelin-1-type A receptor (AT1R and ETAR, respectively) is well described in the pathogenesis of certain medical conditions, suggesting a strong connection between the presence of these antibodies, inflammation, and microvascular function. In systemic sclerosis (SSc), for example, higher levels of autoantibodies against AT1R and ETAR (from now on: AT1R-AAs and ETAR-AAs) are associated with more severe manifestations of disease and predict SSc-related mortality [1]. These findings might be explained by the vasoconstrictor, proinflammatory and profibrotic effects of AT1R-AAs and ETAR-AAs [2,3], which could contribute to SSc pathophysiology. Moreover, among SSc patients, the presence of AT1R-AAs and ETAR-AAs can predict the development of pulmonary arterial hypertension (PAH) and its associated mortality, suggesting the ability of these autoantibodies to increase vascular endothelial reactivity and to induce pulmonary vasculopathy [4]. As a second example, the roles of AT1R-AAs and ETAR-AAs have been deeply studied in the field of transplantation. In kidney transplantation, AT1R-AAs are associated to and involved in the pathogenesis of vascular rejection [5,6], and both AT1R-AAs and ETAR-AAs are associated with graft injury and graft loss [7], supposedly through the impairment of endothelial repair [8]. Similarly, in lung transplantation, AT1R-AAs and ETAR-AAs are associated with antibody-mediated rejection [9], and in liver transplantation, they increase the risk of death, rejection, and allograft fibrosis progression [10,11]. Interestingly, in liver transplantation, the presence of these autoantibodies is also associated with a progressive native renal dysfunction [12], suggesting that AT1R-AAs and ETAR-AAs exert their effects in both transplanted and non-transplanted organs. The effects of AT1R-AAs and ETAR-AAs are also described in severe acute respiratory syndrome coronavirus 2 (COVID-19), in which their titers are significantly increased in patients with an unfavorable disease course [13]. This reflects the effects of AT1R-AAs and ETAR-AAs on endothelial dysfunction, which plays an important role in COVID-19 disease progression [14]. As a fourth and final example, AT1R-AAs and ETAR-AAs are also present in end-stage cystic fibrosis, supposedly as a consequence of prolonged inflammation and deregulated immune response [15].

**Figure 1:**
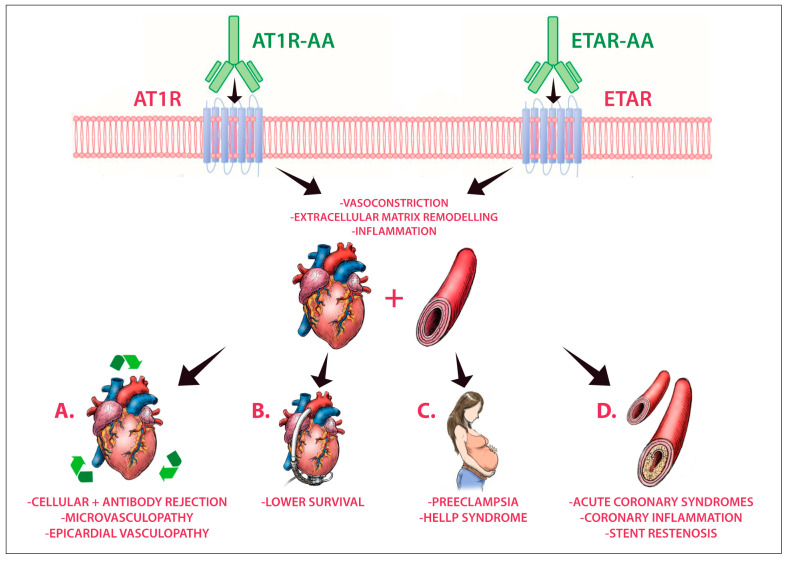
Angiotensin II receptor type 1 (AT1R) and endothelin-1 receptor type A (ETAR) are G-protein coupled-receptors (GPCRs) that are physiologically activated by angiotensin II and endothelin 1, respectively. AT1R and ETAR can also be activated by functional circulating autoantibodies (AT1R-AAs and ETAR-AAs, respectively) that promote vasoconstrictor, profibrotic, and proinflammatory responses. In heart transplant recipients (panel **A**), the presence of AT1R-AAs and ETAR-AAs is associated with graft microvasculopathy, epicardial coronary artery vasculopathy and with cellular- and antibody-mediated rejection. In patients with mechanical circulatory support (panel **B**), a higher prevalence of AT1R-AAs is reported, and it is associated to lower survival. AT1R-AAs are also present in almost all preeclamptic patients, while ETAR-AAs only appear in more severe stages of disease (panel **C**). As regards patients with acute coronary syndromes (panel **D**), higher levels of AT1R-AAs are reported, and their presence is associated with coronary inflammation and stent restenosis.

Although so widely described in many other fields, few data are reported about the roles of these autoantibodies in cardiovascular pathologies. Nevertheless, the influences of AT1R-AAs and ETAR-AAs are of utmost relevance in many cardiac diseases (Figure 1), and their vasoconstrictor and proinflammatory effects on coronary microvascular circulation could be the missing piece to puzzles that we are still not able to finish.

## 2. Angiotensin II (AngII)

AngII is the final effector of the renin–angiotensin–aldosterone system (RAAS) and exerts its effects at the tissue level by stimulating angiotensin-II-type-I and angiotensin-II-type-II receptors (AT1R and AT2R, respectively), by increasing sympathetic tone and vasopressin release and by inducing aldosterone production. The production of AngII depends on an enzymatic cascade of which the first step is renin. Renin, an aspartyl protease, cleaves angiotensinogen to form angiotensin I. Angiotensin I is further cleaved by angiotensin-converting enzyme (ACE) to produce AngII, the physiologically active component of the system that acts on AT1R and AT2R [16].

Most of the known effects of AngII are mediated by AT1R, a G-protein-coupled receptor (GPCR) that is located in many different organ and tissues, including liver, brain, lung, heart, kidney and vasculature. The phosphorylation of serine/threonine residues of AT1R’s cytoplasmic tail by G protein receptor kinase activates the cascade of signal transduction, and even single-nucleotide polymorphisms of the AT1R gene are linked to an increased risk of cardiovascular risk factors, such as hypertension [17], coronary artery disease [18] and myocardial infarction [19]. The major role of AT1Rs in cardiovascular pathophysiology is explained by a higher receptor density in vascular smooth muscle cells (VSMCs), endothelial cells and myocardial cells. The main effects of AngII on these tissues include inflammation, vasoconstriction, fibrosis, lipid oxidation, endothelial dysfunction, expression of adhesion molecules and myocyte hypertrophy [20,21]. Due to these plethora of effects, the expression of AT1R on the cell membrane is closely regulated, and even if in an acute setting increased levels of AngII lead to an increased AT1R activation, chronic stimulation results in receptors downregulation [22,23,24]. AT2Rs take also part in AT1R’s regulation, exerting antiproliferative and proapoptotic actions [25]. AT2R antagonizes AT1R by activating tyrosine or serine/threonine phosphatases that inhibit its signaling [26,27], for example, the deletion of AT2Rs promotes neointimal formation and vascular inflammation [28]. Moreover, AT2R agonists decrease organ fibrosis in in vivo preclinical models [29].

## 3. Endothelin (ET) 1

The ET system consists of two GPCRs—ETAR and ET type B receptor (ETBR)—and three endogenous ligands—ET-1, ET-2 and ET-3. The production of ET-1, the most common form of ET, is initiated in the endothelium by preproET-1 synthesis, which is further cleaved to proET-1. ProET-1 is then proteolyzed to bigET-1, an inactive precursor of ET-1. ET-converting enzyme finally cleaves bigET-1 to ET-1 [30]. ETAR is highly expressed in VSMCs and promotes their contraction and proliferation [31,32]. Vice versa, ET-1 binding to ETBR activates vasodilators production (such as nitric oxide) and facilitates ET-1 clearance from circulation [33,34]. ET-1, first known as a pure vasoconstrictor [35], is implied in the pathogenesis of different cardiovascular diseases, and its role in the pathophysiology of ischemia-reperfusion injury is well established. ET-1 levels, for example, increase in the first hours after myocardial infarction [36], inducing a decrease in microvascular reflow that reduces the benefits of prompt coronary artery reperfusion. The administration of ETAR antagonists therefore improves postischemic microvascular reflow and prevents an increase in myocardial wall thickness [37]. The effects of ET on coronary microcirculation were also studied in animal models of unstable angina, where the blockade of ETAR reduces cyclic coronary flow reduction [38]. Furthermore, ET-1 plays a major role in pulmonary hypertension: there is a significant correlation between the level of ET-1 and the severity of disease [39,40], and ETAR antagonists are the first line drugs in this field.

## 4. AT1R-AAs and ETAR-AAs

AT1R-AAs and ETAR-AAs are autoantibodies directed against AT1R and ETAR, respectively [41]. Their prevalence is well described among patients waiting for transplantation, in whom it ranges between 15% and 40% [42,43], while it is reported to be around 10–15% in small groups of healthy subjects [44,45]. In addition, children born from mothers who have complicated pregnancies express high levels of AT1R-AAs [46]. However, the co-existence of different cut-off levels makes it difficult to compare the prevalence reported in different studies.

AT1R-AAs and ETAR-AAs are functional agonist autoantibodies which are able to activate their target receptors [41]. Their binding results in similar effects to those triggered by natural ligands, including vasoconstriction, extracellular matrix remodeling and proinflammatory cascades [47,48]. However, differently from natural ligands, the binding of these autoantibodies is more sustained, leading to a prolonged activation. AT1R-AAs, for example, induce a 10 times longer vasoconstriction compared to AngII [47,49]. Moreover, while AngII equally activates AT1R and AT2R, resulting in a modulation of AT1R effects as seen above, AT1R-AAs do not activate AT2R, resulting in an unregulated activation of AT1R. ETAR-AAs also bind to ETAR, but not to ETBR, resulting in similar unopposed ETAR activation [47,48].

The genesis of these autoantibodies is still not completely understood, but the analysis of their titers during pregnancy [50] and before and after organ transplantation [51] gives mechanistic insights about factors leading to their production. It was first proposed that a damaged endothelium (both of the host and the graft), with the subsequent shedding of the extracellular portions of AT1R and ETAR, could induce the development of autoantibodies [42]. In addition, end-stage organ disease [52], cystic fibrosis [15], and mechanical circulatory support implantation [53] can activate their production.

## 5. Towards Cardiovascular Pathologies

In the general population, higher serum AT1R-AAs levels strongly correlate with a higher blood pressure, worse functional measures (such as a weaker grip strength and a lower walking speed) and higher rates of falls. Moreover, it was found that even a little increase in AT1R-AAs levels could decrease the time to death, also after adjustment for age, sex, and body-mass index. High levels of AT1R-AAs seem therefore to represent an independent risk factor for adverse outcomes. Interestingly, chronic treatment with angiotensin receptor blockers is associated with a reduction of these adverse outcomes, with an attenuation of the decline in grip strength and an increase in the time to death [54].

Given the physiological effects of angiotensin on blood pressure control, the role of AT1R-AAs in hypertensive disorders has been deeply investigated. Fu et al. first showed the presence of AT1R-AAs in patients with malignant hypertension, suggesting that these autoantibodies might be involved in the pathogenesis of the disease [55]. A hint toward the role of AT1R-AAs in the pathogenesis of hypertension was also given by Liao et al., who found increased titers of autoantibodies in patients with hypertension compared to those of normotensive controls. Moreover, AT1R-AAs were mainly present in patients with refractory hypertension [56]. Higher levels of AT1R-AAs were also present in patients affected by essential hypertension, where their presence was for the first time related to a genetic background (the HLA-DRB1*04 allele) [57].

Following these results in hypertensive disorders, AT1R-AAs were also studied in primary aldosteronism (PA), as they can stimulate aldosterone secretion and trigger the development of a hyperplastic transformation in the zona glomerulosa. Patients with PA were reported to have higher titers of AT1R-AAs compared to those with primary hypertension, and among PA patients, those with aldosterone-producing adenomas had higher autoantibodies levels compared to those with idiopathic hyperaldosteronism [58]. Even if it is still not clear whether AT1R-AAs are the cause or consequence of PA [59], it has been found that AT1R-AAs levels do not normalize after hyperaldosteronism treatment [60]. Interestingly, patients with aldosterone-producing adenomas and women with preeclampsia have comparable levels of AT1R-AAs [58].

### 5.1. Preeclampsia

Preeclampsia is a systemic disease characterized by the onset of hypertension, proteinuria, and endothelial dysfunction during pregnancy [61,62]. It affects 2–8% of all pregnancies and is a leading cause of maternal morbidity and mortality [63]. AngII was long believed to be involved in the pathogenesis of preeclampsia, as AngII infusion in pregnant patients could induce hypertension [64,65]. However, circulating levels of AngII were not increased, and the presence of AT1R stimulating antibodies was demonstrated [66] in almost the totality of preeclamptic patients [50,67]. It was later shown that AT1R-AAs could induce preeclampsia through tissue factor production [68], reactive oxygen species formation [69], and the promotion of an hypercoagulable state [70]. The first in vivo evaluation of the consequences of AT1R-AAs in pregnancy was performed by Zhou et al. [71]. They injected AT1R-AAs from either normotensive pregnant women or pregnant women with preeclampsia in pregnant mice, showing that autoantibodies from women with preeclampsia could induce gestational hypertension and proteinuria in mice. Moreover, autoantibodies injection in nonpregnant mice caused an increase in the blood pressure. All these effects were prevented by co-injection with losartan, suggesting that the functional agonistic role of AT1R-AAs in AT1R could be blocked by AT1R antagonists [50,71]. Based on these findings, it has been suggested that preeclampsia could be an autoimmune disease induced by pregnancy [72].

In addition to AT1R-AAs, in approximately one half of preeclamptic patients, ETAR-AAs have been isolated, and their presence was associated with an higher severity and an earlier presentation of disease [50]. At an experimental level, ETAR-AAs isolated from these patients could induce negative chronotropic response in rate cardiomyocytes, but these effects were blocked by BQ123, a specific ETAR antagonist [50,67]. ETAR antagonists could indeed be useful in blocking the negative effects elicited by ETAR-AAs.

Preeclampsia is also a useful model to study the timing of autoantibodies development. Buttrup Larsen et al. measured the development of antibodies titer in pregnant patients with hypertensive disorders (gestational-induced hypertension, preeclampsia or HELLP syndrome) [50]. Both AT1R-AAs and ETAR-AAs were not present in serum samples collected in the first trimester but developed later in relation to clinical symptoms. In detail, AT1R-AAs developed concurrently with the onset of gestational-induced hypertension or moderate preeclampsia, while ETAR-AAs developed at the stage of severe preeclampsia diagnosis. In the control group of healthy pregnant women, neither ETAR-AAs nor AT1R-AAs were detected.

### 5.2. Heart Transplantation and Mechanical Circulatory Support (MCS)

Similar to kidney [6,7,51] and lung [9] transplantation, the roles of AT1R-AAs and ETAR-AAs have been investigated in heart transplant recipients. Given the ability of these autoantibodies to induce endothelial activation [2,3] and the central role of endothelial activation in allograft vasculopathy [73], it has been hypothesized that the presence of AT1R-AAs and ETAR-AAs could have detrimental effects on allograft function [74]. Heart transplant recipients who experienced any grade of acute cellular or antibody-mediated rejection had higher titers of AT1R-AAs and ETAR-AAs compared to patients who did not develop rejection. Moreover, coming to coronary involvement, 67% of the patients with high autoantibodies levels (cutoff value: 16.5 U/L) developed biopsy-proven microvasculopathy, while this complication was detected in only 23% patients without autoantibodies. Higher autoantibodies titers were also associated with a higher degree of epicardial vasculopathy. Interestingly, in this cohort of patients, those on angiotensin receptor blockers had a lower incidence of microvasculopathy.

As regards MCS, Zhang et al. reported the prevalence of AT1R-AAs in patients before and after MCS implantation. The concentrations of AT1R-AAs increased significantly postimplantation, and among patients with normal titers before implantation, 68% of them reached the saturated concentration (≥40 U/mL) postimplantation [53]. These data are consistent with those reported by Urban et al. [75], presumably due to MCS-associated shear stress that generates neoantigens which stimulate antibody production. AT1R-AAs are also associated with lower survival after MCS implantation, but statistical significance is reached only when assessing survival at 18 months post-implantation; thereafter, the difference of survival is no longer significant [53].

### 5.3. Acute Coronary Syndromes

Important studies have proved the detrimental effects of AT1R-AAs and ETAR-AAs in endothelial repair [8], but their roles in coronary endothelium and function is far from being elucidated. Li et al. analyzed at a molecular level the effects of AT1R-AAs on inflammatory pathways and their potential role in acute coronary syndromes [45]. Patients with an acute coronary syndrome were found to have higher titers of AT1R-AAs compared to both the stable coronary disease group and the healthy control group. As regards inflammation, AT1R-AAs promoted inflammation through the expression of interleukin-6 (IL6), vascular cell adhesion molecule-1 (VCAM-1), and improved levels of NF-kB, a transcription factor that regulates many genes involved in inflammation. These effects were blocked by valsartan. Given these findings, it was hypothesized that AT1R-AAs could promote atherosclerosis (and consequent plaque ruptures and acute coronary syndromes) by inducing vascular inflammation.

Starting from this pathophysiological hypothesis and from the evidence that AT1R blockers (such as valsartan) reduce the incidence of restenosis after coronary stenting [76,77], it has been hypothesized that AT1R-AAs could stimulate the proliferation of VSMCs, neointima hyperplasia and, eventually, stent restenosis. In a group of patients with unstable angina, the prevalence of AT1R-AAs was higher compared to in healthy controls (35.8% vs. 10.2%), and it increased significantly after stent implantation (47.4%). Isolated AT1R-AAs could induce VSMCs proliferation, confirming the hypothesis of their pathogenetic role in neointima hyperplasia and stent restenosis. Moreover, valsartan could markedly inhibit this effect [44]. However, no clear explanation was given about the reason why patients had higher titers of AT1R-AAs after stent implantation.

Further studies are needed in order to expand our knowledge of the effects of AT1R-AAs and ETAR-AAs in acute coronary syndromes and atherosclerosis.

## 6. The Beginning of a New Era?

AT1R-AAs and ETAR-AAs are functional autoantibodies that bind to AT1R and ETAR [41], and their prevalence ranges between 10% and 40%, depending on the population used for sampling [42,43,44,45]. AT1R-AAs and ETAR-AAs mimic the effects of the natural ligands of these receptors (AngII and ET-1, respectively), eliciting vasoconstrictor, profibrotic, and proinflammatory effects [47,48]. In the general population, their presence is associated with worse blood pressure control and increased frailty, representing an independent risk factor for death and adverse outcomes [54]. While their role in solid organ (specifically kidney) transplantation is well described and understood [6,7,47,51,78], the evidence regarding non-alloimmune diseases is scarce. Even in the field of cardiovascular pathologies, the best understanding of the role of these autoantibodies is reached in a disease which is on the edge of alloimmunity—preeclampsia. Almost all preeclamptic patients have abnormal titers of AT1R-AAs, while many of those with an higher severity of disease also have ETAR-AAs [50,67]. AT1R-AAs and ETAR-AAs also display their negative effects in heart transplantation, where they are associated with an higher degree of graft microvasculopathy and epicardial vasculopathy [74]. As regards epicardial coronary artery vasculopathy, AT1R-AAs are implied in vascular inflammation and subsequent atherosclerosis: patients with an acute coronary syndrome have higher titers of these autoantibodies, suggesting that they could be a marker of high risk plaques and atherosclerosis progression [45]. AT1R-AAs are also associated with stent restenosis through the induction of VSMCs and neointima hyperplasia [44].

As the RAAS and the ET system are key factors in cardiovascular pathophysiology, it is not surprising that AT1R-AAs and ETAR-AAs are implied in cardiovascular pathologies. Their systemic vasoconstrictor and proinflammatory effects could play a central role in many cardiac diseases in which vasoconstriction and inflammation are pivotal, from arterial pulmonary hypertension and atrial fibrillation to heart failure with preserved ejection fraction and coronary microvascular dysfunction. However, the current evidence is scarce, and further studies are needed to fill this gap.
